# Determinants of aggression against all health care workers in a large-sized university hospital

**DOI:** 10.1186/s12913-020-05084-x

**Published:** 2020-03-16

**Authors:** Elena Viottini, Gianfranco Politano, Giulio Fornero, Pier Luigi Pavanelli, Paola Borelli, Marco Bonaudo, Maria Michela Gianino

**Affiliations:** 1 San Giovanni Bosco Hospital, ASL Città di Torino, P.zza Donatore di Sangue, 3, 10154 Turin, Italy; 2 Department of Control and Computer Engineering, Politecnico di Torino, Corso Duca degli Abruzzi, 24, 10129 Turin, Italy; 3 Dipartimento Qualità e Sicurezza dei percorsi di Diagnosi e Cura, AOU Città della salute e della Scienza Teaching Hospital, corso Bramante, 88, 10126 Turin, Italy; 4 Sicurezza Ed Ambiente (S.P.P.), AOU Città della salute e della Scienza, Teaching Hospital, corso Bramante, 88, 10126 Turin, Italy; 5 Department of Public Health Sciences and Pediatrics, Università di Torino, Via Santena 5 bis, 10126 Turin, Italy

**Keywords:** Aggression, University hospital, Risk factors, Workplace violence, Verbal and non-verbal aggressive acts

## Abstract

**Background:**

The paper aims to describe the 3-year incidence (2015/17) of aggressive acts against all healthcare workers to identify risk factors associated to violence among a variety of demographic and professional determinants of assaulted, and risk factors related to the circumstances surrounding these events.

**Methods:**

A retrospective observational study of all 10,970 health workers in a large-sized Italian university hospital was performed.

The data, obtained from the “Aggression Reporting Form”, which must be completed by assaulted workers within 72 h of aggression, were collected for the following domains: worker assaulted (sex, age class, years worked); profession (nurses, medical doctors, non-medical support staff, administrative staff, midwives); aggressive acts (activity type during aggressive acts, season, time and location of aggressive acts); and type of aggressive acts (verbal, non-verbal, consequences, aggressors).

**Results:**

Three hundred sixty-four (3.3%) workers experienced almost one aggression. The majority of the assaulted workers were female (77.5%), had worked for 6/15 years and were Nurses (64.3%). The majority of aggressive acts occurred during assistance and patient care (38.2%), in the spring and during the afternoon/morning shifts and took place in locations where patients were present (47.3%). The most prevalent aggression type was verbal (76.9%). The patient was the most common aggressor (46.7%). 56% of those assaulted experienced interruptions in their work.

Being female, being < 50 years of age, having worked for 6–15 years were significant risk factors for aggression. Midwives suffered the highest risk of experiencing aggression (RR = 12.95).

The risk analysis showed that non-verbally aggressive acts were related to assistance and patient care with respect to activity type, to the presence of patients and during the spring and afternoon/evening.

**Conclusions:**

The findings suggest the parallel use of future qualitative studies to clarify the motivation behind aggression. These suggestions are needed for the implementation of additional adequate prevention strategies on either an organizational or a personal level.

## Background

Violence against health workers is a global problem widely described in the literature; it constitutes a risk not only to the dignity but also to the health of workers.

The workplace violence was defined by the National Institute of Occupational Safety and Health (NIOSH) as “the act or threat of violence, ranging from verbal abuse to physical assault directed toward persons at work or on duty.” [26]. There are two types of violence: physical, which involves the use of or the threat of the use of physical force against individuals and includes beatings, kicks, slaps, stabbings, shootings, shoves, and bites; and psychological violence, which includes verbal abuse, rude behaviour, disrespect, an attitude of abuse, intimidation, bullying, harassment, and biting [2].

The International Labor Organization (ILO) has introduced a distinction between “external violence”, which refers to an assault that occurs between employees and any other person present in the workplace (patients, family members, visitors, suppliers) and “internal violence”, which occurs among all classes of workers [24].

According to the Occupational Safety and Health Administration (OSHA), approximately 75% of the almost 25,000 assaults that occur annually have taken place in the health and social services sector [29]. Data collected by the Bureau of Labor of Statistics (BLS) show that from 2002 to 2013, health workers were four times more likely than private industry workers to be victims of violence [30] and that most of these events have a non-fatal outcome; indeed, in 2015, only five fatal cases were recorded [5].

The most common type of aggression is verbal [14, 32, 39], and in the literature, it is highlighted that verbal violence is perpetrated mainly by patients, family members and companions/friends; in contrast, aggressive acts of a physical nature are more often committed by patients with cognitive impairment [43], psychiatric disorders and histories of drug and alcohol abuse [9, 16, 27]. The aggressive acts more frequently involve nurses and non-medical support staff workers and, to a lesser extent, doctors and other professionals [3, 13, 18]. More controversial are the results by sex and age: in some studies, men are more exposed to verbal violence [9], and the risk of aggression is reduced with age [40]; in other studies, greater exposure was observed among women (nurses and doctors) [18], and in smaller contexts, no statistically significant differences were found for sex and age [14].

The violence reflects negatively not only on the person involved, with consequences such as anger, anxiety and anguish, guilt, shame and post-traumatic stress disorder [11, 21], but also on its operations and health organization; indeed, the negative effects of the violence can manifest themselves in the organization’s well-being, highlighting situations of burnout [8], intentions to leave and absenteeism [42].

At the regulatory level, the Member States of the European Community have implemented Directive 89/391 through appropriate legislation, developing guidelines for the prevention of violence at work, and on April 26, 2007, they signed the Framework Agreement, which condemns all forms of harassment and violence and confirms the duty of the employer to protect workers against such risks [4].

In November 2007, the Ministry of Health in Italy published Recommendation n. 8, “Preventing acts of violence against health workers”; this recommendation not only foresees the reporting of incidents of violence using official sources, including reports to the judicial authority or the police or accident reports to Italian National Insurance Institute for Workplace Injuries (INAIL), but also promotes the activation of specific surveys to identify the frequency and severity of episodes, which is useful for the adoption of organizational, structural and training measures that can prevent violence and/or mitigate its negative consequences [25].

The few studies conducted in Italy confirm the presence of the problem of aggression [6, 42, 46, 49].

However, these studies aiming to detect violent attacks against health professionals have used one questionnaire concerning the experience of violence at work; health professionals have been invited to help develop this questionnaire and anonymously describe the worst aggressive acts recorded, usually during the previous 12 months. In addition, these studies focused on certain job categories, such as nurses and physicians [6, 21, 49], and investigated some variables related to the aggressive event, such as location (i.e., patient room, elevator, corridor, waiting room) or activity that preceded the event (i.e., treatment, patient transfer, examination). Finally, these studies analysed cases of assault in medium-sized and general hospitals [6, 46].

The aims of this study are as follows: (i) to describe the 3-year incidence of aggressive acts against all healthcare workers in a large-sized Italian university hospital (consisting of general and specialized hospitals), exploring health professionals’ experience with aggression utilizing a form completed within 72 h of the aggressive act; (ii) to identify potential risk factors associated to aggressive acts among a variety of demographic and professional determinants of healthcare workers assaulted; (iii) to identify potential risk factors for the type of aggressive acts, including activity type during aggression, season of aggression, time of aggression, location of aggression, consequences for victims of aggression and types of aggressors.

## Methods

A retrospective observational study was performed.

The study was carried out in the University Hospital Città della Salute e della Scienza of Turin (AOU). The AOU is one of the largest university hospitals in Europe, and at the national level, it has approximately 11,000 employees, 1917 ordinary hospital beds and 401 day hospital and day surgery beds. The AOU ensures diagnosis and tertiary health care in multiple care paths, favouring multidisciplinary approaches that ensure high-quality and appropriate care. It consists of a general hospital with highly specialized skills (Molinette Hospital) and three specialized hospitals (Child Hospital-Regina Margherita, the Gynecological Obstetric Hospital-Sant’Anna and the Traumatological Hospital-CTO).

This study analysed aggression data from the three-year period of 2015–2017 that included all worker categories.

The data were obtained from the “Aggression Reporting Form”, adopted in 2014 by AOU in compliance with the Recommendation n. 8/2007 of the Ministry of Health. The form is available to all workers on the AOU’s INTRANET portal and must be completed in all its parts by assaulted individuals and sent within 72 h of the aggressive event to the Safety and Environment Office. For this study all participant data were de-identified prior to data collection by the Safety and Environment Office and, consequently, the need for consent was waived by the ethics committee.

Data were collected for the following domains:

Worker assaulted: sex, age class (< 30 years, 30–39 years, 40–49 years, > = 50 years), years worked (<=5 years, 6–15 years, > 25 years), and profession (nurses, medical doctors, non-medical support staff, administrative staff, midwives);

Aggression: activity type during aggression (support activity for patients (i.e., conversation, meal preparation and administration), professional team’s back-office activity (i.e., briefing-debriefing, treatments prescriptions, deliveries for shift change), assistance and patient care, season of aggression (Jan-Mar, Apr-Jun, Jul-Sep, Oct-Dec), time of aggression (night, morning, afternoon, evening), and location of aggression (communal location (i.e., elevator, corridor, bathroom), location in the presence of patients (i.e., patient room, examination room), other activity location (i.e., help desk, doctor room, complaint office));

Type of aggression (verbal and non-verbal): verbal aggression includes insult, offense, and threat, while non-verbal aggression includes violent physical gestures, such as slaps or shoves, the launching of objects, or the use of weapons or blunt instruments;

Aggressor: patients, relatives, caregivers, and colleagues; and.

Consequences of aggression: medical and psychological treatment, interruptions in work, and none.

In this domain we leaved out analysing the most frequently used conditions investigated (department/health units) and we focused on organizational factors less analysed, such as location of aggression, type and time of aggression, activity type during aggression, social and epidemiological factors of the aggressors and assaulted.

To analyse the data, a custom-designed computational pipeline was built using the R framework (R [33]).

Risk analysis was computed using *epiR* [28] and *meta* [41] packages.

For each observation, the pipeline computed the risk ratio with its 95% confidence intervals and its overall Fisher’s *p*-value. In fact, to obtain more reliable results, we performed the risk analysis computation with a Fisher’s exact test for count data to better assess the overall robustness of further risk analysis computations and to be more confident regarding the statistically significant claims, further discussed in the Results section.

The study protocol was approved by the Ethics Committee (November 26, 2018).

## Results

We collected 632 acts of aggression, and 364 workers experienced almost one act of aggression.

The majority of the assaulted workers were female (77.5%) and had worked for 6 to 15 years. The assaulted workers did not differ by age class, and only younger workers (< 30 years old) experienced less aggression.

Among workers who had experienced aggression, nurses reported the highest frequency of violence (64.3%), followed by non-medical support staff (14.4%), whereas administrative staff accounted for the lowest frequency (3.8%). The highest number of aggressive acts occurred during assistance and patient care (38.2%) and support activities for patients (33.8%) and took place in the presence of patients (47.3%), such as in patient rooms and examination rooms, and in communal locations (34.3%). The most frequent events occurred in the spring, between April and June, and during the afternoon and morning shifts.

The most prevalent type of aggression was verbal (76.9%) with insult and offense. The patient was the most common aggressor (46.7%), followed by parents (42.3%), whereas colleagues represented only 3% of aggressors.

A total of 56% (203) of those who were assaulted required working interruption, but only 11.3% (41) of them required medical and psychological treatment.

Detailed characteristics of the workers assaulted, the aggressive acts and the aggressors are summarized in Table 1.

**Table 1:** Characteristics of the workers assaulted, the aggressive acts and the aggressors

Characteristics of the workers assaulted		N	(%)
Workers assaulted (*N* = 364)			
Sex	**Female**	282	(77.5)
	**Unknown**	3	(0.8)
Age classes	**< 30 years**	16	(4.4)
	**30–39 years**	101	(27.7)
	**40–49 years**	124	(34.1)
	**> = 50 years**	118	(32.0)
	**Unknown**	5	(1.4)
Years worked	**<=5 years**	31	(8.5)
	**6–15 years**	177	(48.6)
	**16–25 years**	73	(20.1)
	**> 25 years**	78	(21.4)
	**Unknown**	5	(1.4)
Profession	**Nurse**	234	(64.3)
	**Medical doctor**	23	(6.3)
	**Non-medical support staff**	52	(14.4)
	**Midwife**	39	(10.7)
	**Administrative staff**	14	(3.8)
	**Unknown**	2	(0.5)
Activity type during aggressive acts	**Support activity for patients**	123	(33.8)
	**Professional team’s back-office activity**	70	(19.2)
	**Assistance and patient care**	139	(38.2)
	**Unknown**	32	(8.8)
Time of aggressive acts	**Morning**	119	(32.7)
	**Afternoon**	129	(35.4)
	**Evening**	73	(20.1)
	**Night**	43	(11.8)
Season of aggressive acts	**Jan-Mar**	81	(22.2)
	**Apr-Jun**	118	(32.4)
	**Jul-Sep**	92	(25.3)
	**Oct-Dec**	73	(20.1)
Location of aggressive acts	**Location in the presence of patients**	172	(47.3)
	**Communal location**	125	(34.3)
	**Other activity location**	67	(18.4)
Consequences for victims of aggression	**Medical and psychological treatment**	41	(11.3)
	**Working interruption**	203	(55.8)
	**None**	120	(33)
Characteristics of the aggressive acts and aggressors			
Types of aggression (*N* = 632)			
Verbal	**Insult and offense**	315	(49.8)
	**Threat**	171	(27.1)
Non-verbal	**Violent physical gesture, such as a slap or a shove**	89	(14.1)
	**Launch of objects**	44	(6.9)
	**Use of weapons or blunt instruments**	13	(2.1)
Types of aggressors (*N* = 400)	**Patients**	187	(46.7)
	**Relatives**	169	(42.3)
	**Caregivers**	32	(8)
	**Colleagues**	12	(3)

The results of risk analysis indicated that there is an increased RR of experiencing workplace aggression among females, among workers < 50 years old and among workers with 6–15 years of experience (Table 2).

**Table 2:** Demographic and professional risk factors of healthcare worker assaulted

Variables (Population)	N	RR	CI 95%	*P*-value
Sex				
Female (6814)	282	1.37	[1.08–1.75]	< 0.03
Male (2504)	79	1		
Age class				
< 30 (264)	16	2.63	[1.61–4.30]	< 0.001
30–39 (1254)	101	3.54	[2.76–4.55]	< 0.001
40–49 (2717)	124	1.92	[1.50–2.44]	< 0.001
≥ 50 (5081)	118	1	–	
Years worked				
≤ 5 (1328)	31	1	–	
6–15 (2694)	177	2.8	[1.95–4.03]	< 0.001
16–25 (2708)	73	1.12	[0.74–1.67]	0.528
> 25 (2586)	78	1.26	[0.85–1.88]	0.259
Profession				
Nurse (4177)	234	4.65	[3.07–7.05]	< 0.001
Non-medical support staff (2083)	52	2.04	[1.27–3.29]	0.003
Midwife (248)	39	12.95	[7.96–21.06]	< 0.001
Medical doctor (1894)	23	1	–	
Administrative staff (917)	14	1.37	[0.73–2.58]	0.485

Regarding job categories, midwives suffer the highest risk of experiencing aggression (RR = 12.95), followed by nurses (RR = 4.65) and non-medical support staff (RR = 2.04), in comparison with medical doctors (Table 2).

The risk analysis performed on workers assaulted (Table 3) showed that non-verbally aggressive acts are related to the following:

- Activity type during aggression: individuals providing assistance and patient care have a statistically significant risk of being associated to physical aggression that is 2.27 times higher than that of members of the professional team who perform back-office activities;
- Location of aggression: workers are significantly more exposed in locations in the presence of patients than are workers in locations for other activities;
- Seasons and times: the risk of non-verbally aggressive acts is higher in the April–June period than in the October–December period, and there is a statistically significant risk of being associated to non-verbal assault during the afternoon and evening shifts than during the night shift; and
- Consequences for victims of aggression: non-verbally aggressive acts double the risk of the need for medical and psychological treatment in comparison to no consequences.

**Table 3:** Risk factors related to non-verbal and verbal aggression

Risk factors		Non-verbal aggression					Verbal aggression		
		N	RR	CI 95%	***P***-value	N	RR	CI 95%	***P***-value
**Activity type during aggression**	**Support activity for patients**	21	0.85	[0.46–1.57]	0.698	116	0.97	[0.92–1.03]	0.492
	**Professional team’s back-office activity**	14	1	–		68	1	–	
	**Assistance and patient care**	63	2.27	[1.37–3.75]	< 0.001	122	0.94	[0.88–1.01]	0.226
**Time of aggression**	**Morning**	29	1.50	[0.71–3.16]	0.392	109	1.04	[0.95–1.13]	0.395
	**Afternoon**	42	2.00	[0.97–4.12]	0.050	121	1.01	[0.92–1.11]	1.00
	**Evening**	28	2.36	[1.13–4.93]	0.012	67	0.99	[0.89–1.10]	1.00
	**Night**	7	1	–		40	1	–	
**Season of aggression**	**Jan-Mar**	17	1.02	[0.55–1.90]	1.00	69	0.97	[0.89–1.06]	0.745
	**Apr-Jun**	64	2.64	[1.63–4.27]	< 0.001	108	0.97	[0.90–1.05]	0.573
	**Jul-Sep**	10	0.53	[0.25–1.11]	0.125	91	1.05	[0.99–1.11]	0.171
	**Oct-Dec**	15	1	–		69	1	–	
**Location of aggression**	**Location in the presence of patients**	53	1.20	[0.69–2.10]	0.001	164	1.00	[0.94–1.06]	1.00
	**Communal location**	40	2.19	[1.29–3.71]	0.605	109	0.96	[0.89–1.03]	0.383
	**Other activity location**	13	1	–		64	1	–	
**Consequences for victims of aggression**	**Medical and psychological treatment**	26	1.95	[1.38–2.76]	< 0.001	32	0.81	[0.68–0.95]	< 0.001
	**Working interruption**	41	0.62	[0.43–0.90]	0.016	189	0.99	[0.95–1.04]	1.00
	**None**	39	1	–		116	1	–	
**Types of aggressors**^**a**^	**Patients**	85	3.83	[2.49–5.90]	< 0.001	164	0.93	[0.88–0.98]	0.005
	**Relatives**	16	0.21	[0.13–0.33]	< 0.001	161	1.09	[1.04–1.15]	< 0.001
	**Caregivers**	3	0.30	[0.10–0.90]	0.007	30	1.00	[0.91–1.09]	1.00
	**Colleagues**	3	0.85	[0.32–2.31]	1.00	12	1.06	[1.04–1.09]	1.00

^a^Since each episode may involve multiple type of aggressors, for each attack RR is computed as: the incidental risk of being exposed to a specific aggressor against the exposition to all the other types of attacker

The risk analysis performed on assaulted workers showed that verbally aggressive acts are only related to consequences for victims of aggression, as the victims of verbal aggression had a lower risk of being associated to need medical and psychological treatment than to no consequences (Table 3).

Regarding aggressors, patients had the highest risk (3.83) of engaging in non-verbal aggression, whereas relatives had the highest risk (1.09) of engaging in verbal aggression (Table 3).

## Discussion

The aim of this study was to describe the 3-year incidence of aggressive acts against all healthcare workers in a large-sized Italian university hospital utilizing a form completed within 72 h of the aggressive act to describe important risk factors within this setting. This study confirms existing evidence that aggressive acts are a problem for workers in a large hospital, however the incidence is lower than previous studies [21, 46]: 3.3% of the workers experienced almost one aggression. Several explanations may account for this finding.

First, the perception of some workers on the topic so they did not complete the Aggression Reporting Form and this may have led to underestimation of aggressive acts. A further explanation may lie in the AOU’s prevention policy that have been adopted. Using Aggression Reporting Form data, adopted since 2014, the AOU has conducted a work-site-specific analysis that included an assessment of risk factors associated to aggression acts in specific settings within the hospitals and it has implemented safety measures, such as guards in specific communal location. In addition, AOU’s policy to promote and inform staff of training opportunities that are available to manage both verbal and non-verbal aggressive acts could have reduced cases of overt violence. This study confirms also that verbal violence is the most prevalent form of aggression experienced [1, 13]. Indeed, the number of non-verbally aggressive acts is lower than that of verbally aggressive acts, maintaining a ratio of approximately 1:2/1:3, as indicated in the reviews of Spector et al. and Karen-leigh et al. [17, 43] and in the studies of Kaeser et al. and Terzoni et al. [16, 46].

According to our findings, some demographic and professional variables of workers, such as sex, age, years worked and profession, are significant risk factors associated to aggression.

Female workers are more often victims of violence and are more at risk of aggressive acts than are men. This result is supported by some studies, but findings are not consistent across the literature [6, 20, 42, 49]. Indeed, in some studies, the female sex is associated only with a type of aggression, in particular, with the verbal type [9, 39]; in other studies, however, the variable of interest did not produce statistically significant results [21, 40]. Supported by specialized literature [6, 13, 17, 22, 32, 37, 39, 40, 46], younger and less experienced workers are more at risk of aggression in the workplace than are older (< 50 years old) and more experienced workers. This finding may be attributed to the fact that individuals over 50 years of age are in professional roles, such as those of managers or coordinators, in which contact with patients and relatives is not frequent and is often short in duration. Another possible reason for these results is that more experienced workers have acquired the communication skills and behavioural strategies that are important for preventing or avoiding aggressive acts [13, 15]. A finding in this study is that nurses and non-medical support staff are at high risk of aggression in the workplace. This finding is not surprising and is consistent with those of previous studies [3, 6, 32, 39, 42, 47] and suggests that it is influenced by the fact that nurses and non-medical support staff are the job categories with more frequent contact with patients and relatives. This suggestion is in keeping with findings from previous studies showing differences in the level of aggression experienced by workers depending on the length of time spent with the patient [14, 17, 18, 21, 32]. In addition, considering the type of university hospital in which the study is conducted, aspects of work organization can justify the increased risk of violence against those in job categories such as nurses. Indeed, increases in workload, which are related to low nurse-to-patient ratios caused by nursing shortages and decreased patient length of stay, may lead to violence from patients, since the latter may feel overlooked by the busy nursing staff.

Another important and more unexpected result was that midwives are at the highest risk of aggression. This result has a complex aetiology that may be investigated in the context of the characteristics of the university hospital in which this study is conducted. The AOU includes a gynaecological obstetric hospital that guarantees highly specialized curative and health assistance to women to manage risky pregnancies. This hospital is equipped with an emergency department (ED) addressing all ethnic groups. The highest risk of aggressive acts may be explained either by communication failures, which are a common cause of complaints, and by the presence of language barriers, which impair effective communication. According to an English survey, a significant percentage of women felt that they were neither treated with respect nor spoken to in a way in which they could understand during antenatal and intrapartum care. Women from ethnic minorities and from the most socially deprived group were more likely to have this experience [36]. Moreover, midwives work in the ED, and according to many studies, emergency workers are vulnerable to not only aggressive patients but also relatives or friends [19, 23]. This situation is exacerbated because the women in the gynaecological obstetric hospital’s ED are often victims of violence or sexual violence and are often accompanied by violent companions or family members. Another group not typically discussed in the hospital violence literature, such as administrative staff, was not immune to the aggressive acts, but they were not at significant risk. In the AOU, they are mostly working at the counter providing reservations or giving information to users.

This study, in addition to researching the risk factors related to the characteristics of the workers assaulted, has investigated the aggressive acts to describe the risk factors related to the circumstances surrounding these events, distinguishing different risk factors for different forms of aggression.

It is not surprising that providing assistance and patient care places workers at considerable risk of non-verbally aggressive acts, and it is not unexpected that working in a location in the presence of patients places one at the highest risk of non-verbally aggressive acts, especially if these results are analysed together with the findings on the source of violence that is predominantly performed by patients. Indeed, the literature suggests that hands-on patient care is a risk factor for violence [2, 6, 7, 22, 32, 40, 49] and that non-verbal violence is associated with patients receiving direct care [17, 32].

The highest risk of aggressive acts was reported during afternoon and evening shifts, which is in line with the findings of some studies [37] but not all studies, because the literature also contains discordant findings [45, 46, 48]. The increased risks of assault may be explained, in part, by considering the types of patients admitted to the AOU Città della Salute and by analysing organizational factors. The AOU treats patients who, due to their complexity, require continuous and high assistance during several hours of the day; the AOU treats patients with high expectations; and organizational factors do not guarantee that adequate and qualified staff members are available at all times and require strong shift rotations. Consequently, due the inadequate staffing in the afternoons and evenings versus in the mornings, nurses and non-medical support staff must handle a tremendous workload and amount of stress and prioritize the basic needs of patients. This approach can lead to a lack of effective communication, and some patients’ demands will go unmet, as well as their care expectations, which may lead to patients feeling dissatisfied and engaging in acts of violence [44].

It is more difficult to explain the seasonal risk factors related to non-verbal violence. This topic is not investigated in the literature, and it could be hypothesized that during the winter months, in conjunction with the influence of the peak of overcrowding of the EDs and the organizational suffering of some units, there is an increase in episodes of aggression. Instead, only a greater probability of suffering non-verbal aggression during the spring months emerged. This finding requires further research for clarification.

In terms of consequences, this study highlights that there is a significantly high risk that non-verbally aggressive acts and a significantly low risk that verbally aggressive acts appear associated to medical or psychological care performed in hospital and home settings.

Our study has several strengths.

Several studies utilize questionnaires to explore the experiences of health professionals being subjected to aggressive acts, and most studies use a long time frame, typically a 12-month period [13]. For the 12-month period, the self-assessment could have been affected by recall bias, including the problem of overreporting or underreporting because the long period could influence the memory as well as what was reported and because staff that experienced aggression may have left the workplace and therefore would not have participated in the studies. The methodology used in this study, based on data reported on a form filled out within 72 h of aggression, reduces recall bias [12] and therefore makes some differences (e.g., between medical doctors and other health professionals groups) more explicit. In addition, an elevated risk of patient/visitor perpetrated violence against hospital nurses and physicians has been reported by several studies, while little is known about violence among other hospital workers. This study includes all job categories working in the hospital, thus making the findings more representative of the problem in health care settings. A characteristic of this study that can be read as a strength is that it analyses the incidence of aggression against all healthcare workers in a large university hospital, where the type of patients and organizational variables (i.e., inpatient and acute psychiatric services, geriatric long-term care settings, and high-volume urban EDs) that epidemiological studies consistently demonstrate to be the greatest risk factors are present.

This study also has limits. The first limit is related to a lack of aggressor data, which, if available, may have highlighted particular demographic characteristics, such as sex, migratory background, illnesses and cognitive states of violent patients or particular sociodemographic characteristics, including expectations of the hospital, that may have been useful to identify other potential risk factors. Some studies have shown that violence is a problem experienced by nurses working with migrants or immigrant backgrounds in emergency care [6, 31, 49]. Other studies have shown that aggressive acts arise when patients who believe that they deserve high-quality care and positive outcomes judge the assistance they receive as inadequate and develop public distrust and anger towards healthcare workers [50]. Other studies have highlighted that mentally ill patients or patients under the influence of alcohol or drugs are more common aggressors [9, 25, 27]. Furthermore, the availability of aggressor data may allow to use of models that use multiple factors that impact on violence in the health sector.

In accordance with the narrative review conducted by Ramacciati et al. [34] the synergy of several factors (internal and external from the working environment) explains the incidence of violence in the healthcare sector. This highlights a multidimensional point of view that analyzes theoretical models that reck the physical and psychological conditions of the patient, and organizational factors such as absenteeism, productivity loss and work-related stress.

Finally, although we utilized a database that is subject to specific legislation and regulations, we recognize that the reporting of aggressive acts can be influenced by bias, such as cultural norms within the AOU, personal expectations, education and training in the management of aggression and beliefs regarding obligations and the attribution of blame [10, 38]. This bias, not yet restrained by the AOU’s prevention policy, may result in the underreporting of aggressive acts or the selection of certain types of aggression. This selection is subjective and often only the most serious aggression are reported while the verbal ones are considered part of working life [35].

## Conclusions

This study confirms that aggression is one of the major problems in health care activities, but this paper adds results on what is already known on the topic.

This paper demonstrates that aggression is also a workplace problem in a large-sized university hospital for all job categories of workers, however the incidence is low. In addition, this study identifies potential risk factors associated to aggressive acts among a variety of demographic and professional determinants of assault among healthcare workers, as well as potential risk factors related to the circumstances surrounding these events, distinguishing different risk factors for different forms of aggression.

The tool enables the workers to rapidly and easily report aggression within 72 h anonymously, thus overcoming the problem of recall bias and mitigating the difficulty of gaging the extent of the problem. This latter difficulty is also avoided because all workers in the AOU are involved, rather than merely a sample of workers.

The findings suggest the need for future qualitative studies in parallel to clarify in detail the motivation of aggression and to assess how workers have experienced episodes of violence. These studies are needed to implement additional adequate prevention strategies either on an organizational or on a personal level.

## Data Availability

The datasets used and/or analysed during the current study are available from the corresponding author on reasonable request.

## Abbreviations

AOU: University Hospital
BLS: Bureau of labor of statistics
CDC: Center of disease control and prevention
CGCOM: General council of official medical associations of Spain
CTO: Traumatological Center Hospital
EC: European commission
ED: Emergency department
ILO: International labor organization
INAIL: National workplace accident Institute
NIOSH: National Institute of Occupational safety and health
ONAM: National observatory of aggressions to physicians
OSHA: Occupational safety and health administration
RR: Relative Risk
